# Early Screening Strategies for Diabetes Mellitus by Leveraging Dental Visits Using Optimal Screening Tools Available Onsite

**DOI:** 10.7759/cureus.3641

**Published:** 2018-11-26

**Authors:** Wasique Mirza, Muhammad Sabih Saleem, Gaurav Patel, Pravin Chacko, Sandhya Reddy, Renee Schaefer, Richard Jones, Nisha Dheer, Sirish Dharampuri, Ameet Sandhu

**Affiliations:** 1 Internal Medicine, Geisinger Commonwealth School of Medicine, Scranton, USA; 2 Internal Medicine, Shifa International Hospital, Islamabad, PAK; 3 Cardiology, The Wright Center for Graduate Medical Education, Scranton, USA; 4 Internal Medicine, The Wright Center for Graduate Medical Education, Scranton, USA; 5 Internal Medicine, Scranton Primary Health Care Center, Scranton, USA; 6 Dentistry, Scranton Primary Health Care Center, Scranton, USA

**Keywords:** diabetes, early diagnosis, interdisciplinary approach, diabetes and dental disease

## Abstract

Diabetes mellitus (DM) continues to be a major health concern in the Western hemisphere, and the management of type 2 DM (T2DM) is an ongoing challenge for the American healthcare system despite major advances in DM research. T2DM imparts a massive economic burden, and a major challenge in managing T2DM continues to be timely screening. Adults are more likely to visit a dentist than a primary care physician. This study was designed to compare current screening standards recommended by the American Diabetes Association with the use of gingival-crevicular blood and its compatibility with traditional methods using a fingerstick. Patients routinely presenting to the dental clinic were offered participation in the trial and, after informed consent, checked for blood glucose levels using the fingerstick method as a control. The control values were compared to the results of the gingival-crevicular blood glucose test obtained during the dental procedure from the same patient (i.e., patients were their own controls). A total of 226 study participants were included. Of these, 127 (56.1%) participants were women, whereas 99 (43.9%) participants were men. The sample size was derived using the Slovin’s equation (Power = 80%) statistical test. We used the Pearson coefficient test to measure the statistical difference between the two tests. We found no significant difference in glucose readings between the fingerstick method and the gingival methods of collection (t = -1.134, P = 0.258). A small sample was also tested for glycosylated hemoglobin (HbA1c) using the same sample collecting methods. However, due to the cost restraints involved in using HbA1c kits, a statistically significant cohort could not be collected. By incorporating this interdisciplinary approach, testing for DM during routine dental visits can be a vital resource for the early diagnosis of DM, potentially leading to significant savings in future healthcare costs.

## Introduction

Diabetes mellitus (DM), one of the top three incurable chronic medical conditions, continues to be a major health concern in the Western hemisphere [1]. Despite several decades of important diabetes-related research advances, the management of type 2 DM (T2DM) proves to be a major challenge to the American healthcare system. A recent statement from the American Diabetes Association (ADA) estimated that T2DM accounts for an economic burden of $245 billion, which is a significant increase from earlier figures [2]. A major challenge in managing T2DM continues to be timely screening, with an estimated seven million people going undiagnosed every year [3].

While continuing research for new therapeutic modalities and prevention strategies are essential, developing effective health systems that screen for T2DM, and, hence, diagnose T2DM earlier, will significantly improve glycemic control, prevent chronic morbidities, and, ultimately, mitigate the burden of T2DM on the healthcare system.

According to the ADA, approximately 8.1 million people remained undiagnosed with T2DM in 2012. One of the main barriers to preventing favorable outcomes in T2DM is the lack of routine patient checkups, especially for those in the early phase of the disease. In this regard, several providers have tried to leverage dental office visits as a means to integrate these important checkups into patient’s lives. At least 67.2% of adults visit a dentist for any reason every year compared to 52.2% who visit a primary care physician, according to the 2014 National Ambulatory Medical Guidelines [4]. This study will attempt to compare the current screening standards recommended by the ADA with the use of gingival-crevicular (GC) blood and its compatibility with the traditional fingerstick (FS) methods of blood collection. While previous studies have investigated this relationship with varying results, we incorporated a larger portion of our community to enhance our study. We used office-based glycosylated hemoglobin (HbA1c) laboratory machines for ease of access, quick results to diagnose, and arranged timely follow-ups with a primary care physician [5-6].

Moreover, the patient population of interest in this study is predominantly those patients who are underinsured or uninsured in a Federally Qualified Health Center setting. The early detection and intervention of T2DM in this vulnerable population can be of significant importance for early treatment and the prevention of long-term complications.

## Materials and methods

We selected the routine dental population from the Scranton, Dunmore, Taylor, Dickson City, and Moosic, Pennsylvania, areas. Study participants were selected from a primary health care clinic and a dental clinic. The targeted sample size was calculated using the Slovin’s formula (Power = 80%) statistical test. We used the Pearson coefficient test to measure the statistical difference between the two tests. All adult patients (age ≥18 years) were included in the study. Patients who had suppressed immunity, bacterial endocarditis, a bleeding disorder, an organ transplant or on immunosuppressants therapy, dialysis, prosthetic valves, and those with dental abscesses at the site of sample collection were excluded from the study. We trained dental assistants and resident physicians on the proper methodology, and we obtained informed consent for the screening from all subjects in the dental clinic.

After obtaining consent, FS blood and GC blood were collected, and random blood sugar (RBS) levels from those samples were tested using an Accu-Chek® glucometer (Roche Diagnostics, Indianapolis, Indiana, USA). FS blood was collected via single-use lancets. GC blood was obtained using micropipettes. During the dental procedure, available GC blood was collected and tested for RBS levels and compared to the results of the FS blood sample. All subjects in the study were tested by both methods. Hence, test and control samples were obtained from the same patient. There was no bias in the selection of patients, as the population was obtained from the regular outpatient dental population scheduled by individuals unassociated with the study.

The GC blood samples were collected from blood that is invariably produced during any standard dental procedure. The dental assistants collected the blood samples under the direct supervision of the dentist and resident physicians. The Internal Medicine office was available for assistance and troubleshooting, as needed. We used the Pearson coefficient test to measure the statistical difference between the two tests. In the event of abnormal glucose test results, we followed ADA guidance and performed an HbA1c test simultaneously on FS and GC blood samples. Based on the ADA criteria and time interval since their last meal, patients who were screened positive for T2DM were urged to follow up with a primary care physician (PCP). If the patients did not already have a PCP, a prompt appointment at an Internal Medicine primary care clinic was made via a pre-established channel with the clinic staff. A feedback questionnaire (indexed below) was used to assess patient acceptability of dental visit diabetic screening. Participant data and information were protected via physical and electronic security. Those who did not have a PCP would be offered an appointment at a primary care clinic within a one-month period. Those who already had a PCP were advised to obtain an appointment with their PCP as soon as possible.

## Results

**Figure 1:**
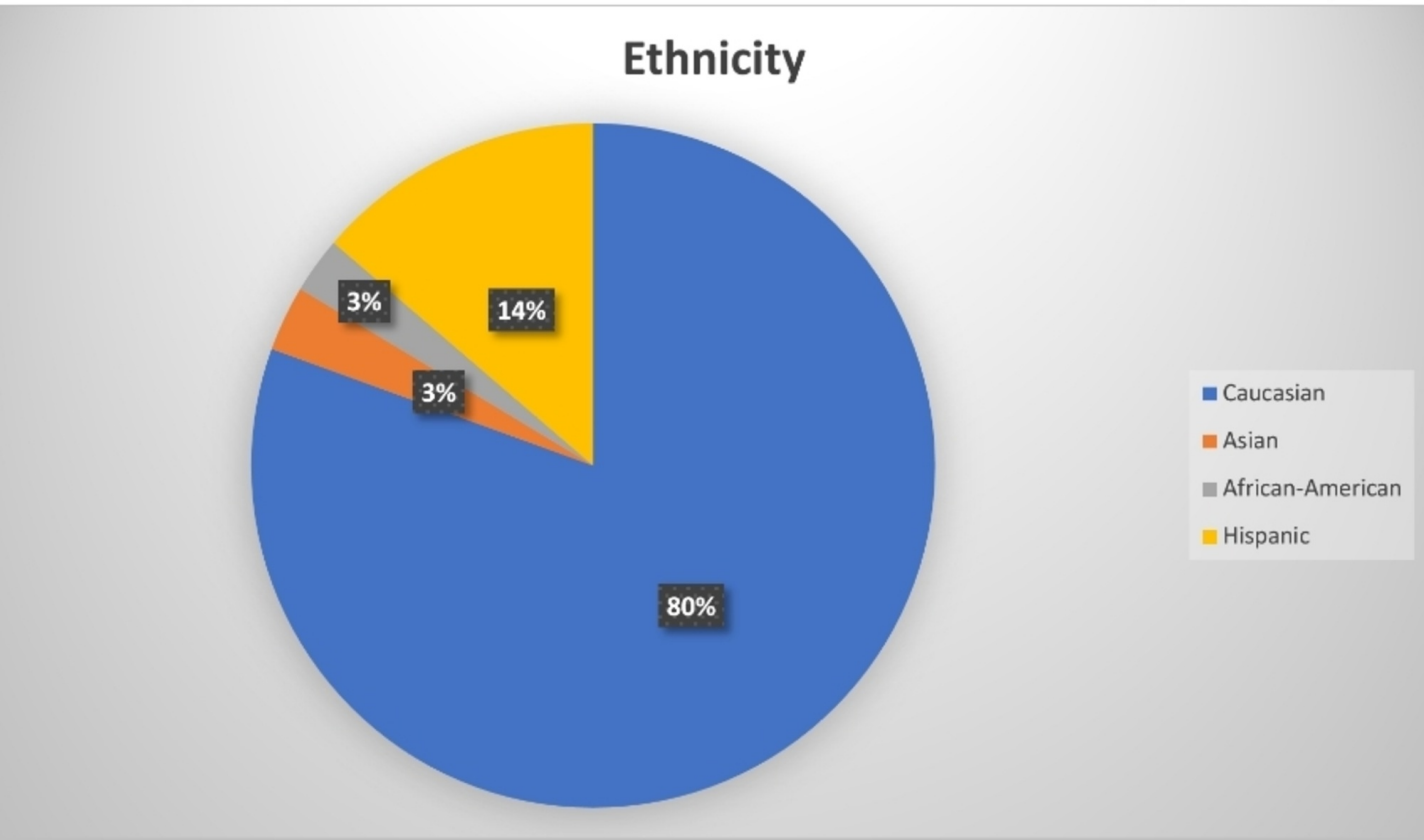
Ethnicity

**Figure 2:**
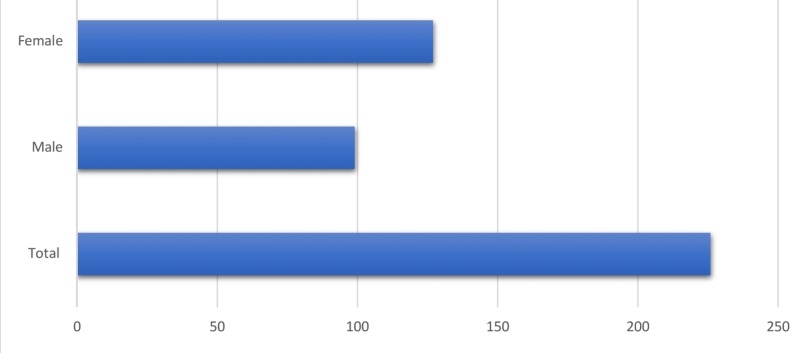
Patient population (gender distribution)

A total of 226 study participants were included in the study. Of these, 127 (56.1%) participants were women and 99 (43.9%) were men. One hundred and eighty-two (80.5%) participants were Caucasians, six (2.7%) participants were African American, seven (3.1%) were Asian, and 31 (13.7%) were Hispanic (see Figures 1-2). All the participants were adults who were able to consent and were above 18 years of age at the time of data collection. With a mean age of 49 years, 24 (10.61%) participants had T2DM and 35 (15.48%) had hypertension. Forty-five (19.9%) glucose readings were collected in fasting state while 181 (80.1%) glucose readings were an RBS measurement. The results seen with both methods were similar, with no significant difference in glucose readings between the FS and GC methods of collection (t = -1.134, P = 0.258) (Table 1).

**Table 1:** Average blood glucose values

Total Patients	Average Fingerstick Blood Glucose	Average Gingival-Crevicular Blood Glucose	Average Age of Patients	p-value
226	109 mg/dl	112 mg/dl	49	0.258

## Discussion

In T2DM, early diagnosis, treatment, and continuous monitoring of blood glucose are linked to a significant reduction in the development of complications. For optimal glycemic control, timing is linked to all-cause mortality. Early diagnosis and treatment are also directly linked to an enhancement in the overall well-being and can lead to a significant reduction in healthcare costs, which was at an estimated $245 billion in 2012 according to the ADA [2]. A significant segment of the population does not engage in preventive health care on a regular basis, which prohibits the early diagnosis and prevention of avoidable complications. Almost two-thirds of the general population visit a dentist’s office for a variety of reasons, including routine cleaning and treatment for a toothache. Introducing means to screen for T2DM at dental clinics can be a means of early detection and diagnosis of T2DM [5-6]. Uncontrolled T2DM is a factor in gum disease development; conversely, untreated gum disease can increase HbA1c levels by as much as 1%. A study from Finland suggested individuals with toothbrush self-efficacy had lower plaque scores and lower HbA1c levels compared to those who had poorer self-efficacy [7-8]. Furthermore, patients with DM who managed their gingivitis successfully tended to report better glycemic control and had a lower HbA1c (8.1 ± 1.5%) compared to those who did not manage their gingivitis [9]. Evidently, the resolution of periodontal inflammation improves metabolic control (with reductions of 0.4% in HbA1c levels), though large, multicenter, randomized controlled trials are needed to further validate these findings [10].

Procedures in dental clinics can be long and tedious. Much instrumentation is required, and the expectation of adding the additional burden of T2DM screening using an FS and glucometer may lead to an additional burden on the dental staff. However, access to a blood sample is not difficult in the dental setting, especially when deep cleaning and extractions are involved.

Our study focused on ways to screen for T2DM in the outpatient settings of dental offices, thus reaching a segment of the population who may not be seen in medical offices until their T2DM is mature and complications have started to present. Instead of a traditional FS with a lancet device, a blood sample was obtained from the GC blood available during the normal course of a dental procedure. We compared the GC readings to the capillary blood readings obtained via FS and evaluated their compatibility. As was shown in a previous, smaller study, the two readings were statistically compatible, suggesting this method may be introduced to dental practices and potentially lead to a dramatically increased scope of screening for T2DM. We obtained samples using the GC method and tested HbA1c from those samples using our office-based system comparing it with FS samples. However, this line of inquiry was limited by the funding restrictions of using the office-based HbA1c kits. A scientific conclusion could not be achieved due to the small sample size. However, the limited results suggested similar trends in the compatibility of results and warrant a further study in this regard.

## Conclusions

Blood samples collected via traditional FS methods yielded statistically similar blood glucose results in GC blood samples generated and collected in a dental setting during a routine dental visit. Introducing these practices to dental clinics will require a concerted effort to educate and introduce a procedure that will require a cultural change. It is a challenging task, albeit one that has the potential to make a big difference. Large-scale studies may be required to validate these findings before such steps are taken. However, if successful, this approach can lead to a paradigm shift in the interprofessional approach towards recognizing and treating diabetes.
